# Highly successful production of viable mice derived from vitrified germinal vesicle oocytes

**DOI:** 10.1371/journal.pone.0248050

**Published:** 2021-03-11

**Authors:** Maki Kamoshita, Katsuyoshi Fujiwara, Junya Ito, Naomi Kashiwazaki

**Affiliations:** 1 Laboratory of Animal Reproduction, Graduate School of Veterinary Sciences, Azabu University, Sagamihara, Kanagawa, Japan; 2 School of Veterinary Medicine, Azabu University, Sagamihara, Kanagawa, Japan; China Agricultural University, CHINA

## Abstract

The vitrification of immature germinal vesicle (GV) oocytes is an important way to preserve genetic resources and female fertility. However, it is well known that cryopreserved GV oocytes have very poor developmental ability and that further improvement in this technique is needed. We previously reported the successful vitrification of matured mouse oocytes with enclosed cumulus cells using the calcium-free vitrification solution supplemented with ethylene glycol (EG) by the minimal volume cooling (MVC) method. In this study, we investigated whether our method is applicable to the vitrification of mouse oocytes at the GV stage (GV oocytes). Following maturation and fertilization *in vitro*, vitrified GV oocytes showed high survival (94.3 ± 2.0%) and maturation (94.3 ± 2.1%) rates. Although the fertilization and blastocyst rates of vitrified oocytes (fertilization: 46.6 ± 4.9% and blastocyst: 46.6 ± 3.0%) were significantly lower than those of fresh oocytes (fertilization: 73.0 ± 7.1% and blastocyst: 71.6 ± 8.0%) (*P* < 0.01), there were no differences in the ability to develop to term between fresh oocytes (50.0 ± 8.4%) and vitrified oocytes (37.5 ± 4.6%) (*P* > 0.05). In conclusion, we here show, for the first time, the efficient production of live mice derived from vitrified GV oocytes.

## Introduction

The cryopreservation of germ cells can contribute to the efficient production of farm livestock and laboratory animals, the gene banking of female resources, and the improvement of human-assisted reproductive technologies. In recent years, oocyte cryopreservation has become more popular as a means of fertility preservation not only for women diagnosed with cancer prior to gonadotoxic therapy but also for women who wish to preserve their oocytes for non-medical reasons [1]. In particular, the cryopreservation of immature oocytes at the germinal vesicle (GV) stage (GV oocytes) has a safety advantage for women because GV oocytes can be collected with fewer gonadotropins, resulting in decreased exposure to excess hormonal levels and a reduced risk of developing ovarian hyperstimulation syndrome and hormone-sensitive malignancies [2]. In addition, oocytes at the metaphase-II (MII) stage are very sensitive to chilling due to their meiotic spindle, whereas GV oocytes have a lower sensitivity to low temperatures than MII-stage oocytes [3], since GV oocytes are arrested at the prophase I stage of meiosis and the spindle has not yet formed in GV oocytes [2]. These facts suggest that the cryopreservation of GV oocytes can be one of the important technologies for the female fertility preservation.

Although the vitrification of mouse GV oocytes already described in 1989 [4], the successful generation of live birth had not been reported until Aono et al. [5], succeeded in first generation in 2005. Aono et al. [5] demonstrated that the 10-step pre-equilibrium method using an obliquely cut 0.25-mL plastic straw with a small droplet (< 1 μL) improved the *in vitro* developmental ability (42.9%) of vitrified mouse GV oocytes as compared with the single pre-equilibrium method (23.7%). However, the rate of live birth was still low (10%) following embryo transfer [5]. In addition, 10-step pre-equilibration is required before vitrification in this method, and thus this method may increase the risk of losing oocytes during the procedure. Thus, there is a demand for a simpler and more efficient protocol of mouse GV oocytes vitrification.

Recently, we have succeeded in improving MII oocytes vitrification by the minimal volume cooling (MVC) method [6] with some modification in mice [7–9] and rats [10]. In these reports, we demonstrated that MII oocytes vitrified with ethylene glycol (EG) as a permeable cryoprotectant in the calcium-free medium showed higher developmental competence as compared to oocytes vitrified with dimethyl sulfoxide (DMSO) and EG in the calcium-supplemented medium [7, 8, 10]. In addition, we showed the importance of cumulus cells for the IVF efficiency of vitrified mouse MII oocytes by comparing cumulus-oocytes complexes (COCs) to denuded oocytes [7, 8]. In results, we achieved a high ability to develop to term after the vitrification of MII oocytes, which generally had shown low developmental ability after IVF [7, 8].

In the present study, we investigated the ability of maturation, fertilization and development of mouse GV oocytes with cumulus cells vitrified using the calcium-free medium supplemented with EG by the MVC method.

## Materials and methods

All chemicals and reagents were purchased from Sigma-Aldrich (St. Louis, MO, USA) unless otherwise stated. The animal study was approved by the Animal Research Committee of Azabu University (Permission Number: 110325–1), and the mice were maintained and sacrificed in accordance with the Azabu University Guidelines for the Care and Use of Laboratory Animals.

### Oocyte preparation

Specific-pathogen-free ICR females (3 to 5 weeks old) were purchased from Charles River Japan (Kanagawa, Japan). The mice were housed in an environmentally controlled room with a 12 h dark (18:00 to 6:00 hours) and 12 h light cycle at a temperature of 23 ± 2°C and a humidity of 55 ± 5% with free access to a laboratory diet and filtered water. ICR females were intraperitoneally injected with 5 IU equine chorionic gonadotropin (eCG: PMS-A 1000 for animals, Nippon Zenyaku Kogyo Co., Ltd., Fukushima, Japan). At 48 h after the eCG injection, mice were sacrificed by cervical dislocation by well-trained individuals and the ovaries were collected into Minimum Essential Medium Alpha (MEMα, no Nucleosides, Powder; Life Technologies, CA, USA) supplemented with 5% fetal calf serum (FCS: Life Technologies, CA, USA) and then GV oocytes with cumulus cells were collected by puncturing antral follicles with a 26-G needle (Top, Tokyo, Japan). The COCs were washed 3 times and kept in the same medium at 37.5°C until they were subjected to treatments.

### In vitro maturation (IVM) of GV oocytes

The suitable duration of maturation *in vitro* in mouse GV oocytes was evaluated. The fresh COCs were cultured in 100 μL droplets of MEMα supplemented with 5% FCS, 10 ng/mL epidermal growth factor (Wako, Osaka, Japan), 2.1 mg/mL sodium bicarbonate, 75 mg/mL penicillin G salts, and 50 mg/mL streptomycin sulfate under liquid paraffin oil (Kanto Chemical, Tokyo, Japan) at 37.5°C in an atmosphere of 5% CO_2_ in air for 8, 10, 12, 14, or 16 h. After the culture, the fresh COCs were used for IVF to evaluate fertilization and developmental ability.

### Vitrification of GV oocytes

Vitrification was carried out by the modified MVC method as previously described [7]. In brief, COCs were washed 3 times with calcium-free modified Dulbecco’s phosphate-buffered medium (PB1) [11] + 20% (v/v) FCS at 37.5°C. The COCs were placed in the equilibration solution composed of 15% (v/v) EG (Kanto Chemical, Tokyo, Japan) + 20% (v/v) FCS in calcium-free PB1 for 3 min at room temperature (25°C). After equilibration, the COCs were exposed to the vitrification solution composed of 30% (v/v) EG + 0.5 M sucrose + 20% (v/v) FCS in calcium-free PB1 at 25°C for 1 min and then vitrified using Cryotop [12]. The COCs were stored in liquid nitrogen (LN) for at least 3 days. The COCs were warmed in 1.0 M sucrose + 20% (v/v) FCS in calcium-free PB1 at 37.5°C for 1 min. The COCs were exposed to the dilution solution composed of 0.5 M sucrose + 20% (v/v) FCS in calcium-free PB1 for 3 min, and then placed in calcium-free PB1 supplemented with 20% (v/v) FCS at 25°C for 5 min. After warming, the COCs were matured for 14 h *in vitro*. As a control, COCs were also collected and then matured *in vitro* without vitrification. After IVM, the fresh or vitrified oocytes were used for IVF as follows.

### IVF and sperm cryopreservation

IVF was performed as described by Kohaya *et al*. [7]. For sperm collection, B6D2F1 males (12 weeks old) were purchased from Charles River Japan (Kanagawa, Japan). Male mice were sacrificed, cauda epididymides were collected at 25°C, and the epididymides were placed in a 35-mm sterile plastic dish containing 400 μL R18S3 (18% (w/v) raffinose and 3% (w/v) skim milk (Wako)) medium [13]. For freezing, R18S3 containing spermatozoa was loaded into 0.25-mL plastic straws (Fujihira Industry, Tokyo, Japan). The straws were exposed to LN vapor for 10 min (about -150°C) and then plunged into LN and stored for at least a week. For thawing, the straws were kept in a 37.5°C water bath for 10 sec, and the contents were then expelled into a 35-mm sterile plastic dish. The frozen/thawed sperm were incubated in TYH medium [14] to induce capacitation at 37.5°C under 5% CO_2_ in air for an hour, and then the thawed sperm were added to TYH medium drops containing matured oocytes (final sperm concentration was 0.2 × 10^6^ sperm/mL) and co-cultured at 37.5°C for 6 h. After the culture, the COCs were washed 3 times in KSOM-AA medium [15]. The cumulus cells of oocytes were removed by gently pipetting, and then the survival of the oocytes was morphologically evaluated. The maturation and fertility of the oocytes were also evaluated using a *Hoffman* modulation *contrast* microscope (IX70; Olympus, Yokohama, Japan). The maturation of an oocyte was defined as extrusion of a 1st polar body, and fertilized oocytes were determined based on the observation of 2PNs. Only fertilized oocytes were transferred into 50-μL drops of KOSM-AA and cultured up to 96 h at 37.5°C under 5% CO_2_ in air. Two-cell and blastocyst formation were evaluated at 18 h and 96 h post-fertilization, respectively.

### Embryo transfer

After IVF, the 2-cells were surgically transferred into the oviducts of recipients after the induction of pseudopregnancy as described previously [7]. ICR females (8–24 weeks old) as recipients for embryo transfer were mated with vasectomized ICR males (15–50 weeks old) on day 0 between 18:00 and 20:00 to induce pseudopregnancy. Six to nine of the 2-cells embryos were transferred into each oviduct of the recipients on day 1. On day 21, offspring were obtained via natural birth. If offspring had not been delivered through natural birth on day 22, Caesarean section was performed.

### Statistical analysis

Each experiment included at least three replicates. More than 70 oocytes were used for each treatment group in the study. All data were subjected to arcsine transformation before statistical analysis. Statistical analysis was performed by Statcel ver.3 (Add-in software for Microsoft Excel, OMS Ltd., Japan). To evaluate the IVM period of fresh GV oocytes, analysis of variance and Tukey-Kramer’s test were used. The rates of survival, maturation, 2PNs formation, 2-cell, blastocyst formation, and development to term of fresh or vitrified GV oocytes were analyzed by a two-tailed, Welch’s t-test. Differences were considered to be significant at *P* < 0.05. Data are shown as means ± standard errors of the means (SEMs).

## Results

### Effect of IVM periods on maturation, fertilization, and developmental ability in vitro

**Fig 1:**
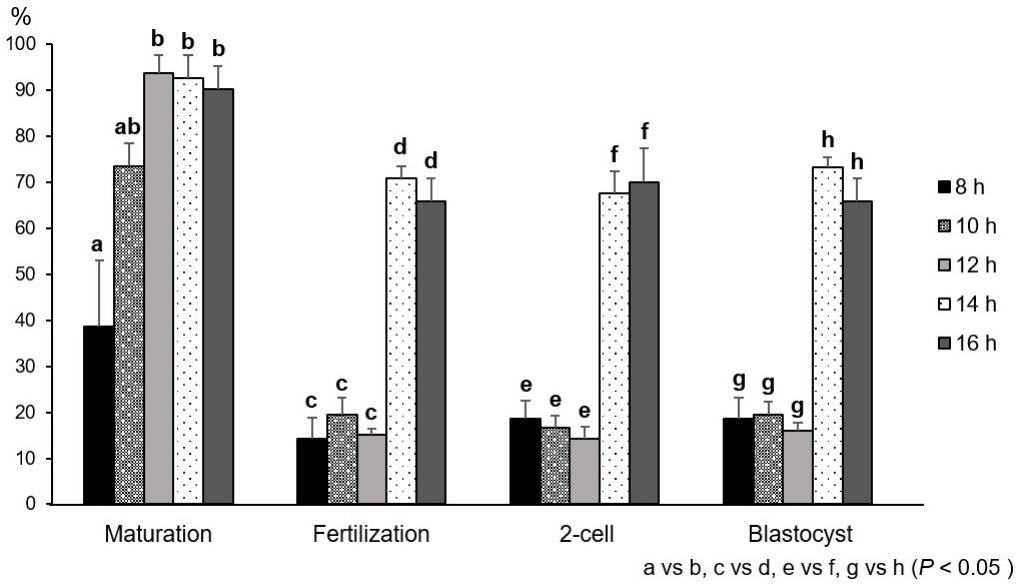
Evaluation of IVM period using fresh mouse oocytes. After IVM and IVF, cumulus cells were removed, and then 1st polar bodies were observed to determine the maturation rate and 2 PNs were observed to determine fertilization. The formation of 2-cell and blastocyst were confirmed following the culture. IVF was carried out using frozen-thawed B6D2F1 mouse sperm. Data are means ± SEM. Different superscripts denote a significant difference (*P* < 0.05). The number of oocytes for the data analysis in each group is as follows: 8 h = 70, 10 h = 72, 12 h = 126, 14 h = 108, and 16 h = 103, respectively.

We first examined the IVM period of vitrification of GV oocytes that was optimal for maturation, fertilization, and developmental ability *in vitro*. Fresh COCs were cultured in IVM media for 8, 10, 12, 14, and 16 h. We confirmed the extrusion of the 1st polar body to evaluate the maturation efficiency and the formation of 2 pronuclei (2PNs) to assess the rate of *in vitro* fertilization (IVF). The rates of the oocytes with a polar body were 93.7 ± 3.9% (12 h), 92.7 ± 5.0% (14 h), and 90.3 ± 5.1% (16 h), which were dramatically higher than that of 38.6 ± 14.3% (8 h) (*P* < 0.05) (Fig 1). At 14 h and 16 h, the rates of 2PNs formation (14 h: 71.0 ± 2.6% and 16 h: 66.0 ± 5.0%) were significantly higher than the rates at the other time points (8 h: 14.3 ± 4.5%, 10 h: 19.4 ± 3.7%, and 12 h: 15.1 ± 1.3%) (Fig 1). In addition, the rates of development to the 2-cell and blastocyst stages were 73.4 ± 2.0% (14 h) and 66.0 ± 5.0% (16 h), which were higher than the rates of 18.6 ± 4.6% (8 h), 19.4 ± 2.9% (10 h), and 15.9 ± 1.8% (12 h), while most of the fertilized oocytes developed to blastocysts (Fig 1). Based on these results, the maturation period of 14 h was used for the following studies.

### Survival, maturation, and fertility of GV oocytes with cumulus cells vitrified with the calcium-free medium supplemented with EG by the MVC method

**Fig 2:**
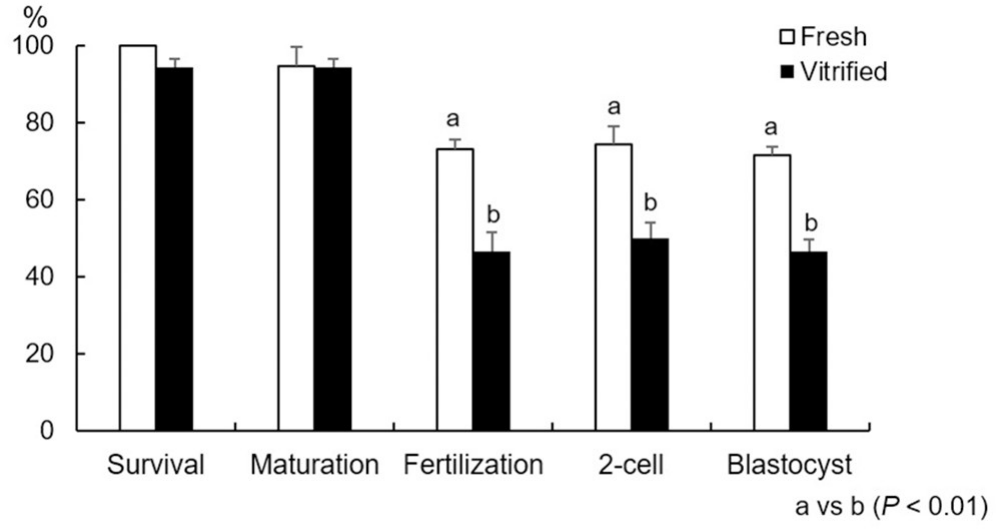
Rates of survival, maturation, fertilization, and development of vitrified oocytes compared with fresh oocytes. IVF was performed after IVM for 14 h. Data are means ± SEM. Different superscripts denote a significant difference (*P* < 0.05). The number of oocytes for the data analysis in each group is as follows: Fresh = 74 and Vitrified = 88, respectively. n.d., no data.

In our previous report, exposure for 3 min in the equilibration solution was adopted for mouse MII oocytes [7–9] and mouse embryos [16]; however, it remains unknown whether 3 min is a suitable equilibration time for GV oocytes. We next assessed the optimal exposure time for GV oocytes. COCs were exposed in the equilibration solution for 3, 5, or 7 min and then vitrified. As a result, we found that the survival, fertilization, and developmental rates of vitrified mouse GV oocytes were not affected by 3, 5, and 7 min exposure times (S1 Fig), and we adopted 3 min as the exposure time for this study. We examined the survival, maturation, and fertility of vitrified GV oocytes compared to fresh oocytes after 14 h IVM and subsequent IVF. The rates of survival, maturation, and fertilization after IVF are shown in Fig 2. The vitrified GV oocytes showed high survival rate (94.3 ± 2.0%). There were no significant differences between fresh and vitrified GV oocytes in maturation rate (94.6 ± 3.6% and 94.3 ± 2.1%) (*P* > 0.05). We observed a decrease in the fertilization rate in vitrified oocytes (46.6 ± 4.9%) compared with fresh oocytes (73.0 ± 7.1%). Although the 2-cell and blastocyst rates were lower in vitrified oocytes (2-cell: 50.0 ± 4.0%; blastocyst: 46.6 ± 3.0%) than in fresh oocytes (2-cell: 74.3 ± 7.5%; blastocyst: 71.6 ± 8.0%), most fertilized oocytes developed to the 2-cell and blastocyst stages following vitrification.

### Vitrified GV oocytes showed a high ability to develop to term

**Fig 3:**
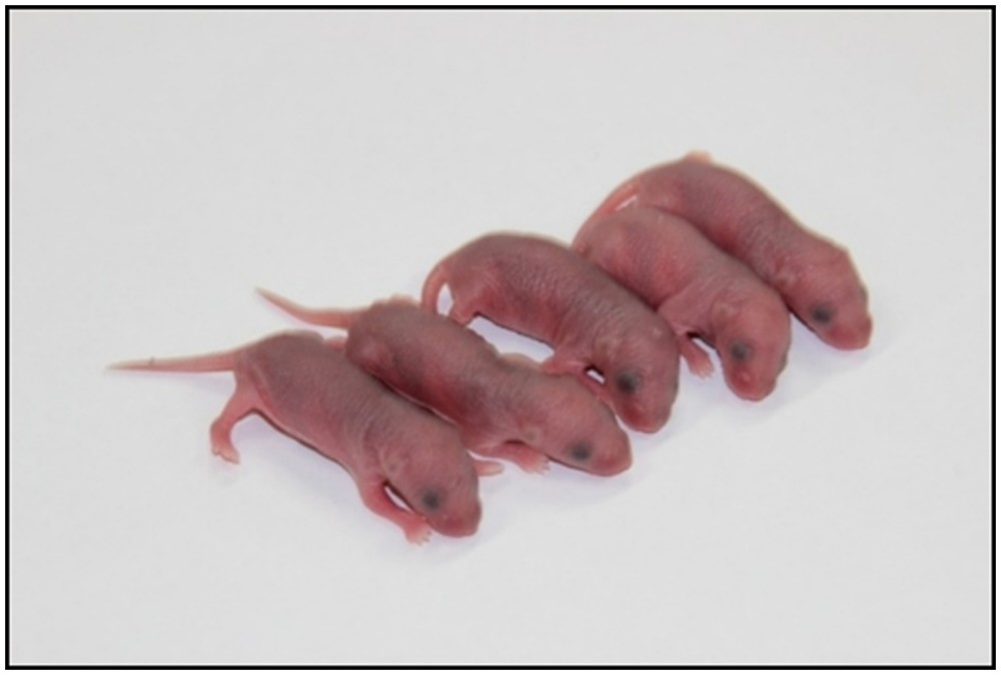
A representative image of offspring by transferring of the 2-cells delivered from vitrified GV oocytes. Vitrified/warmed oocytes were matured and fertilized *in vitro* and then cultured until the 2-cell stage. The embryos were transferred into pseudopregnant mice.

Based on the results of the *in vitro* study, we further examined whether vitrified GV oocytes can be developed to term. Vitrified/warmed COCs were cultured to the 2-cell stage *in vitro* after IVM and IVF. The 2-cells were then transferred into pseudopregnant females. In the fresh oocytes group, 72 embryos were transferred to 5 recipients, and 36 pups were delivered (50.0 ± 8.4%), whereas in the vitrified oocytes group, 88 embryos were transferred to 5 recipients, and 33 pups were obtained (37.5 ± 4.6%) (Table 1). There were no significant differences in development to term between the fresh and vitrified groups (*P* > 0.05). All pups were morphologically normal and healthy (Fig 3).

**Table 1:** *In vivo* development of 2-cells delivered from vitrified mouse oocytes after IVM and IVF.

Oocyte status	No. of transferred embryos	Pregnancy/transfer (%)	Offspring (%)
Fresh	72	5 / 5 (100)	36 (50.0 ± 8.4)
Vitrified	88	5 / 5 (100)	33 (37.5 ± 4.6)

There were no significant differences (*P* > 0.05).

## Discussion

In the present study, we successfully produced mice that were delivered from vitrified GV oocytes. For decades, the cryopreservation of mouse GV oocytes has achieved high survival and maturation rates *in vitro* by various methods and approaches; by slow freezing [17] and by vitrification using Cryotop [18–20], Open pulled straw [21], EM grid [22], and Cryoloop [23], however, the fertilization and developmental abilities are still lower than those in fresh oocytes. In particular, there have been no reports that succeeded in generating live offspring since Aono *et al* [5] published. Aono *et al*. [5] previously showed the successful production of live mice delivered from vitrified GV oocytes by the 10-step pre-equilibrium method for the first time. They achieved a high blastocyst rate (42.9%) from vitrified GV oocytes following IVM and IVF. Likewise, we achieved a high blastocyst rate (46.6 ± 3.0%) delivered from vitrified COCs. In addition, our study demonstrated that the embryos delivered from vitrified GV oocytes developed to term with a high success rate (37.5 ± 4.6%) as compared to that of the 10-step pre-equilibrium method (about 10%). The rate was not significantly different from that obtained with fresh GV oocytes (50.0 ± 8.4%). Although the 10-step pre-equilibrium method seems to show a similar rate of development to blastocysts, this method requires 10 steps before vitrification as mentioned above. Conversely, our method used in the present work can be simply performed and reduces the amount of steps.

High *in vitro* developmental abilities of mouse GV oocytes after vitrification were obtained by both the 10-step pre-equilibrium method [5] and our method used in the present work. In contrast, the ability to develop to term of oocytes vitrified with our method was better than that of the 10-step pre-equilibrium method. One possible reason for this is the absence or presence of calcium and DMSO in the vitrification solution. In our previous reports, we also demonstrated that the calcium-free medium supplemented with EG dramatically improved the fertility, and the ability to develop to term of vitrified oocytes was approximately equal to that of fresh oocytes [7, 8]. EG has less toxicity than DMSO [24], which is used as a permeable cryoprotectant combined with EG in the 10-step pre-equilibrium method [5]. Moreover, EG can help suppress the increase of intercellular calcium [25], which is related to the exocytosis of cortical granules and induces zona hardening [26]. Based on these findings, we suggested that the medium without calcium and supplemented with EG is suitable for the vitrification of mouse MII oocytes. However, the role of an increase in intercellular calcium in GV oocytes was still unclear. More recently, Wakai and Fissore [27] showed that a calcium leak constitutively occurs from the endoplasmic reticulum, and this calcium leak ceases around the resumption of meiosis from the GV stage. They also found that mitochondria absorb calcium during the calcium oscillations, and the mitochondrial redox and increase of ATP production are stimulated by the calcium oscillations in GV oocytes [27]. These findings suggest that extracellular calcium from a general vitrification solution might interfere with intercellular calcium homeostasis and result in low maturation and fertility after vitrification; therefore our calcium-free medium supplemented with EG is suitable not only for mouse MII oocytes but also for GV oocytes.

Another possibility is that our MVC method is more appropriate for mouse GV oocyte vitrification than the 10-step pre-equilibrium method. To date, the MVC method using Cryotop [12] has been used for the vitrification of oocytes in several species including bovine oocytes [28], ovine oocytes [29], rat oocytes [10], and porcine oocytes [30]. These reports suggest that the MVC method by Cryotop [12] would be one of the most effective vitrification methods for mammalian oocytes. In 2015, Abedpour and Rajaei [18] demonstrated the vitrification of mouse GV oocytes by the MVC method using Cryotop; however, the fertilization and blastocyst rates were still low (44.1% and 20%, respectively). The reason that the fertilization and blastocyst rates were low might have been that they removed cumulus cells before vitrification. Previously, we reported that vitrified cumulus oocyte complexes (COCs) could preserve high fertility in mouse oocytes at the MII stage [7, 8]. In line with these reports, Lee et al. [19] showed that the maturation percentage after GV oocyte vitrification was significantly higher in the COC than in denuded oocytes. It is well known that cumulus cells communicate with the oocyte by gap junctions. Gap junctions are necessary for oocytes to resume meiosis and acquire cytoplasmic maturation and subsequently developmental competence [31]. Cumulus cells are also important for fertilization because chemokines secreted from cumulus cells induce sperm capacitation and enhance fertilization [32]. Vincent et al. [33] also demonstrated that the presence of cumulus cells minimizes the release of cortical granules in mice. Accordingly, there is no doubt that minimizing the disruption of cumulus cells by handling during oocyte collection and cryopreservation is essential to maintaining fertility after cryopreservation of the GV oocytes in mice. For these reasons, GV oocytes vitrified by our method might achieve a strong ability to develop to term.

In conclusion, the present study demonstrated that mouse GV oocytes were efficiently vitrified with cumulus cells using the calcium-free medium supplemented with EG by the MVC method. The present report could contribute to efficient mouse production, and to the further development of human-assisted reproductive technologies.

## Supporting information

S1 Fig The effect of exposure time during vitrification on survival, maturation, fertilization and development. Data are means ± SEM. ANOVA and Tukey-Kramer’s test were used for quantification. There are no differences among the group (P > 0.05). The number of oocytes for the data analysis in each group is as follows: 3 min = 88, 5 min = 97, and 7 min = 101, respectively.
(TIF)
