# Supplementary figures and images for: Highly successful production of viable mice derived from vitrified germinal vesicle oocytes
Source: PLoS One. 2021 Mar 11;16(3):e0248050. doi: 10.1371/journal.pone.0248050 (PMC7951897; doi:10.1371/journal.pone.0248050)

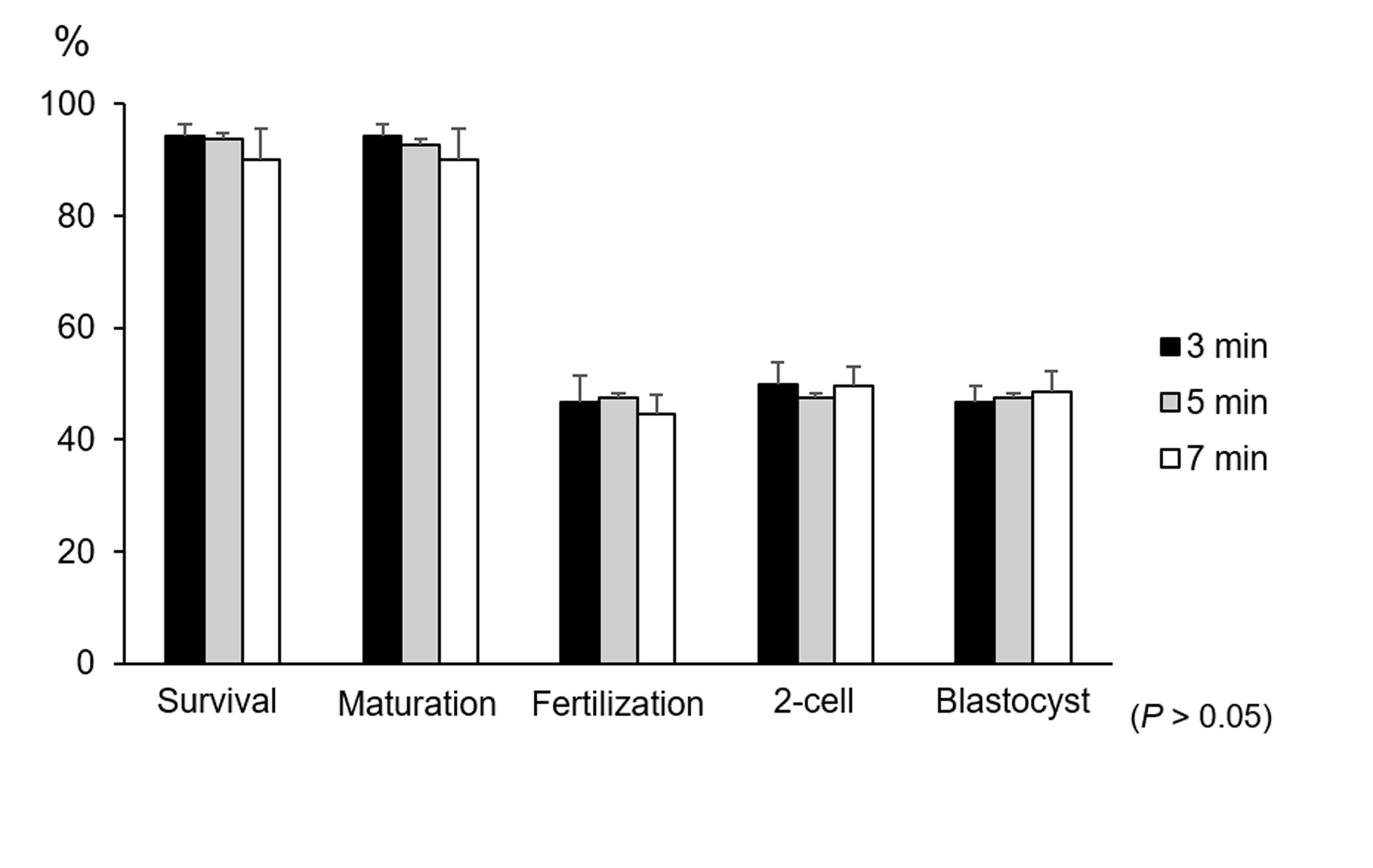

Supplement: S1 Fig — Data are means ± SEM. ANOVA and Tukey-Kramer’s test were used for quantification. There are no differences among the group (P > 0.05). The number of oocytes for the data analysis in each group is as follows: 3 min = 88, 5 min = 97, and 7 min = 101, respectively. (TIF) [file pone.0248050.s001.tif]
